# Left ventricular remodeling and hypertrophy in patients with aortic stenosis: insights from cardiovascular magnetic resonance

**DOI:** 10.1186/1532-429X-14-50

**Published:** 2012-07-28

**Authors:** Marc R Dweck, Sanjiv Joshi, Timothy Murigu, Ankur Gulati, Francisco Alpendurada, Andrew Jabbour, Alicia Maceira, Isabelle Roussin, David B Northridge, Philip J Kilner, Stuart A Cook, Nicholas A Boon, John Pepper, Raad H Mohiaddin, David E Newby, Dudley J Pennell, Sanjay K Prasad

**Affiliations:** 1 Royal Brompton Hospital, Sydney Street, London, SW3 6NP, United Kingdom; 2 Centre for Cardiovascular Science, Edinburgh University, Edinburgh, United Kingdom; 3 Imperial College London, London, United Kingdom

**Keywords:** Aortic valve disease, MRI, Cardiac remodeling

## Abstract

**Background:**

Cardiovascular magnetic resonance (CMR) is the gold standard non-invasive method for determining left ventricular (LV) mass and volume but has not been used previously to characterise the LV remodeling response in aortic stenosis. We sought to investigate the degree and patterns of hypertrophy in aortic stenosis using CMR.

**Methods:**

Patients with moderate or severe aortic stenosis, normal coronary arteries and no other significant valve lesions or cardiomyopathy were scanned by CMR with valve severity assessed by planimetry and velocity mapping. The extent and patterns of hypertrophy were investigated using measurements of the LV mass index, indexed LV volumes and the LV mass/volume ratio. Asymmetric forms of remodeling and hypertrophy were defined by a regional wall thickening **≥**13 mm and >1.5-fold the thickness of the opposing myocardial segment.

**Results:**

Ninety-one patients (61±21 years; 57 male) with aortic stenosis (aortic valve area 0.93±0.32cm2) were recruited. The severity of aortic stenosis was unrelated to the degree (r^2^=0.012, P=0.43) and pattern (P=0.22) of hypertrophy. By univariate analysis, only male sex demonstrated an association with LV mass index (P=0.02). Six patterns of LV adaption were observed: normal ventricular geometry (n=11), concentric remodeling (n=11), asymmetric remodeling (n=11), concentric hypertrophy (n=34), asymmetric hypertrophy (n=14) and LV decompensation (n=10). Asymmetric patterns displayed considerable overlap in appearances (wall thickness 17±2mm) with hypertrophic cardiomyopathy.

**Conclusions:**

We have demonstrated that in patients with moderate and severe aortic stenosis, the pattern of LV adaption and degree of hypertrophy do not closely correlate with the severity of valve narrowing and that asymmetric patterns of wall thickening are common.

**Trial registration:**

ClinicalTrials.gov Reference Number: NCT00930735

## Background

Aortic stenosis is characterised by progressive narrowing of the aortic valve and can be considered the paradigm for left ventricular pressure overload. The ventricle responds to this pressure overload by triggering a hypertrophic response, leading to an increase in myocyte size, left ventricular wall thickness and mass. Initially this response restores wall stress [1,2] but ultimately proves maladaptive and predicts an adverse prognosis both in the context of hypertension and aortic stenosis [3-5].

There is wide individual variation in both the degree and pattern of hypertrophy observed in aortic stenosis. Indeed, four patterns of anatomic adaption have been described in response to an increased afterload on the basis of echocardiographic measurements of left ventricular mass, volumes and the relative wall thickness [6,7]. These patterns are: *normal ventricular geometry*, c*oncentric remodeling*, c*oncentric hypertrophy,* and *eccentric hypertrophy.* Asymmetric patterns have also been reported [8,9], however these have not been included in the above or other definitions.

The assessment of left ventricular remodeling and hypertrophy by echocardiography has several limitations when compared to cardiovascular magnetic resonance (CMR). This latter technique offers the more precise measurements of left ventricular mass, volume and wall thickness [10,11] but has not previously been used to characterise the hypertrophic response of the left ventricle in aortic stenosis. The aim of this study was to use CMR to investigate both the different morphological patterns of LV adaption observed in this condition and the factors affecting the magnitude of the hypertrophic response.

## Methods

### Patients with aortic stenosis

Patients referred to the Royal Brompton Hospital CMR Unit between January 2003 and November 2009 with moderate or severe aortic stenosis on their most recent echocardiogram (peak velocity >3 m/s, aortic valve area <1.5 cm^2^) were studied. In our institution, local guidelines recommend CMR for all patients with severe aortic stenosis. Other reasons for referral included diagnostic evaluation, clarification of disease severity, pre-operative evaluation, and assessment of the hypertrophic response. In order to study the effects of aortic stenosis on the ventricle in isolation, our cohort was carefully selected to avoid patients with confounding drivers of left ventricular remodeling. Exclusion criteria therefore included those with prior myocardial infarction, uncontrolled hypertension (>180/120 mmHg), significant valve disease other than aortic stenosis (moderate or severe mitral, tricuspid or pulmonary valve disease and moderate or severe aortic regurgitation), a clinical diagnosis of co-existent cardiomyopathy including hypertrophic cardiomyopathy, amyloidosis, disseminated malignancy and severe renal failure (estimated glomerular filtration rate <30 mL/min). Coronary artery disease was excluded by invasive coronary angiography (84% of the cohort), multidetector computed tomography coronary angiography (8%) or stress perfusion imaging (2%). In patients under 40 years, it was excluded in the absence of symptoms or risk factors (6%). Comprehensive baseline clinical characteristics and history were obtained using a standardised structured proforma and were completed from source clinic record data and patient questionnaires. The study was conducted with the patient’s consent, local research ethics committee approval and in accordance with the declaration of Helsinki.

### CMR protocol

CMR was performed on Avanto 1.5 T magnetic resonance scanners (Siemens, Erlangen, Germany) using steady-state free precession sequences for the assessment of left ventricular volumes and mass. Aortic valve stenosis severity was assessed by echocardiography and confirmed by CMR using a combination of peak velocity and planimetry of the aortic valve area [12]. For quantification of left ventricular function and volumes, endocardial and epicardial contours were identified in end-diastole and end-systole, and planimetry performed with dedicated semi-automated analysis software (CMR Tools, Cardiovascular Imaging Solutions, London, UK). The left ventricular mass was calculated from the total myocardial volume multiplied by the specific gravity of the myocardium (1.05 g/mL), with trabeculations and papillary muscles included. The left ventricular mass and volumes (end-diastolic and end-systolic) were indexed to body surface area (calculated using the Mosteller formula) to provide the LV mass index and indexed volumes. Left ventricular dilatation and hypertrophy were defined as an indexed left ventricular end-diastolic volume (LVEDV) and mass >95^th^ percentile of the widely-used normal range, published by Maceira et al, corrected for age and gender [13]. Similarly left ventricular ejection fraction was reduced if below the 95^th^ percentile [13]. The maximal wall thickness was measured by blinded observers from the short-axis views of the left ventricle in end diastole, and its position with reference to the 17-segment model of the left ventricle recorded [14]. Right and left ventricular trabeculations were excluded from wall thickness measurements. Asymmetric left ventricular wall thickening was defined as a regional wall thickening ≥13 mm that was also >1.5-fold the thickness of the opposing myocardial segment. Criteria had to be fulfilled on two adjacent short-axis slices.

### The LV mass/volume ratio (M/V)

The LV mass/volume ratio (M/V) is calculated by dividing the left ventricular mass by the left ventricular end diastolic volume [15-17]. It indexes wall thickness to cavity size and is therefore the conceptual equivalent of the echocardiogram-derived relative wall thickness (twice the posterior wall thickness divided by the LV end-diastolic diameter). However, because M/V lacks a well-defined normal reference range, age and sex-matched healthy volunteers without co-existent coronary artery disease, hypertension, aortic stenosis or other forms of heart disease were recruited and scanned contemporaneously. The normal range calculated from this cohort was then applied to the patients with aortic stenosis.

### Definition of the patterns of left ventricular hypertrophy and remodeling

**Figure 1:**
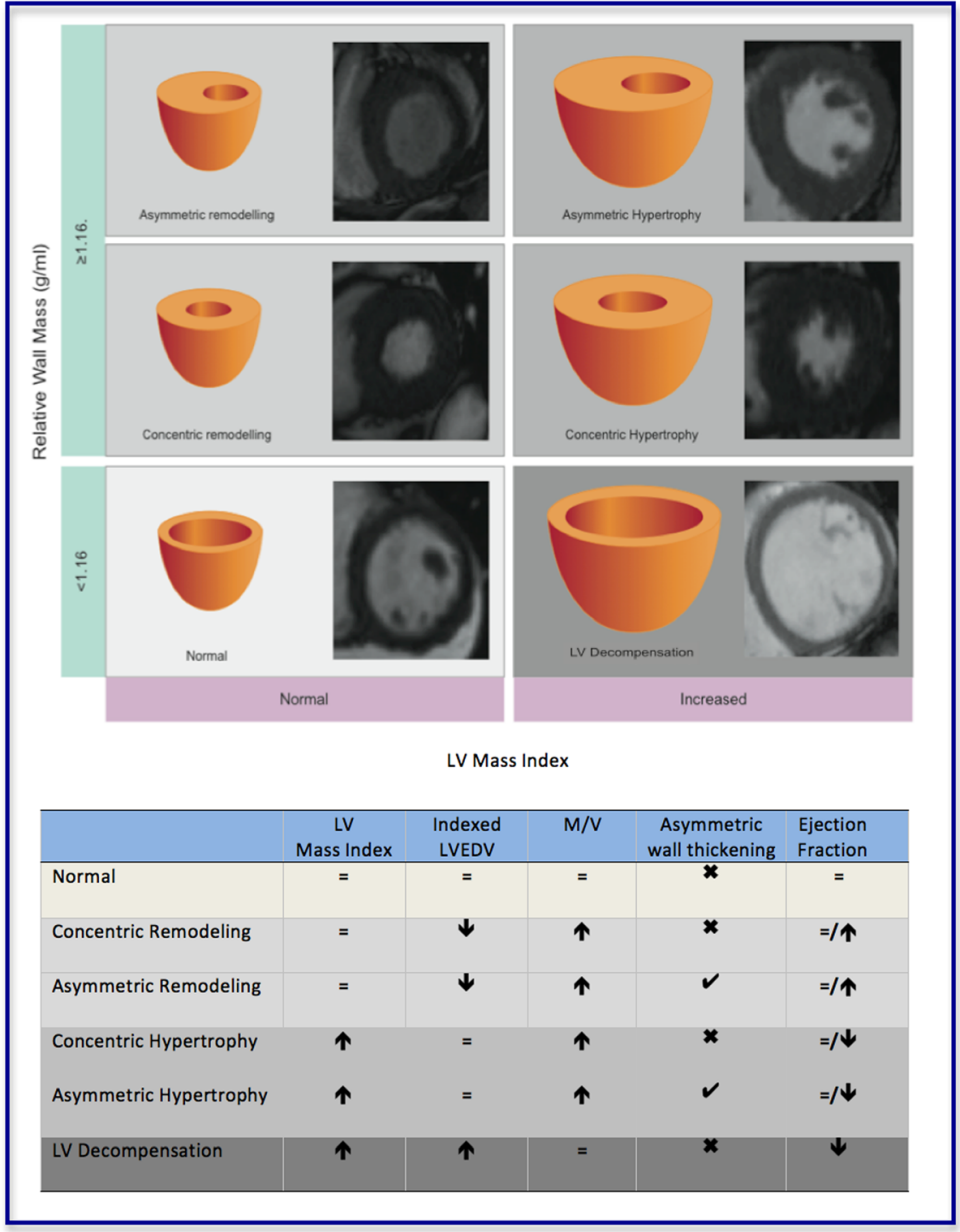
**CMR definitions of the six patterns of left ventricular hypertrophy and remodeling in aortic stenosis.** Schematic representation of the left ventricular structure alongside CMR short-axis images of the left ventricle in end-diastole. ***Normal ventricular structure:*** characterised by a normal LV mass index, indexed LVEDV, and a normal M/V. ***Concentric remodeling:*** characterised by an increased M/V and normal LV mass index. ***Asymmetric remodeling:*** similar to concentric remodeling except that in addition there is evidence of asymmetric wall thickening. ***Concentric Hypertrophy:*** characterised by an increased M/V and LV mass index. ***Asymmetric hypertrophy:*** similar to concentric hypertrophy except that in addition there is evidence of asymmetric wall thickening*.****Left Ventricular Decompensation:*** characterised by a dilated left ventricle and normal M/V. The LV mass index may be increased primarily due to LV dilatation. Note no patients fulfilled the criteria for eccentric hypertrophy and so this was replaced by LV decompensation. **↑**increased; **↓**decreased; = normal; ✓present; ✖absent.

Aortic stenosis patients were categorised into six, pre-defined patterns of left ventricular anatomic adaption according to the LV mass index, the indexed LVEDV and M/V as follows. **Normal ventricular structure**: characterised by a normal M/V, normal LV mass index and normal indexed LVEDV. **Concentric remodeling**: characterised by an increased M/V but normal LV mass index. **Asymmetric remodeling**: similar to concentric remodeling but with evidence of asymmetric wall thickening. **Concentric Hypertrophy**: characterised by an increased M/V and LV mass index. **Asymmetric hypertrophy**: similar to concentric hypertrophy but with evidence of asymmetric wall thickening. **Eccentric hypertrophy**: characterised by an increased LV mass index, a dilated left ventricle, normal M/V and a normal ejection fraction (Figure 1).

### Statistical analysis

Continuous variables were expressed as mean ± standard deviation, and compared using unpaired Student’s *t*-test or one-way analysis of variance where appropriate. Categorical variables were expressed as percentages and analyzed using the chi-squared test or Fisher’s exact test as appropriate. Statistical significance was defined as two-sided P < 0.05. Correlation between normally distributed data was performed using Pearson’s correlation to provide r^2^ values. Univariate analysis was performed to determine independent predictors of LV mass index. Further sub-group analyses were performed in patients with and without concomitant hypertension, and according to sex. All statistical analysis was performed using Stata 10.1 software (StataCorp, Texas, USA).

## Results

### Study population

Ninety-one patients (61 ± 21 years; 57 male; 37 with a history of hypertension) were assessed and had moderate (31%) or severe (69%) aortic stenosis and a mean aortic valve area (AVA) of 0.93 ± 0.32 cm^2^ (Table 1). Ninety-one healthy control subjects were identified and matched to the aortic stenosis cohort for age and sex (mean age 61 ± 10 years, 61% male). M/V in this group was 0.88 ± 0.14 g/mL (95% confidence intervals: 0.60 to 1.16), consistent with previous studies [16]. There was no correlation between M/V values and age (r^2^ = 0.04, P = 0.073). The M/V was subsequently calculated for each patient with aortic stenosis and values above 1.16 g/mL were considered elevated.

**Table 1:** Characteristics of patients with different forms of remodeling and hypertrophy

	**Normal ventricle**	**Concentric remodeling**	**Asymmetric remodeling**	**Concentric hypertrophy**	**Asymmetric hypertrophy**	**LV decompensation**	**P value**
Number	11	11	11	34	14	10	-
Male sex (%)	45	55	82	65	64	60	0.62
Age (years)	52 ± 26	54 ± 21	70 ± 12	57 ± 18	75 ± 11	69 ± 18	0.01*
CMR DATA							
Indexed LVEDV (mL/m^2^)	76 ± 9	55 ± 12	56 ± 9	77 ± 19	78 ± 24	126 ± 34	<0.01*
LV mass index (g/m^2^)	63 ± 11	75 ± 10	78 ± 7	113 ± 21	110 ± 24	106 ± 18	<0.01*
M/V (g/mL)	0.84 ± 0.16	1.39 ± 0.31	1.43 ± 0.28	1.51 ± 0.28	1.47 ± 0.33	0.88 ± 0.19	<0.01*
Maximal wall thickness (mm)	11 ± 2	13 ± 3	17 ± 2	15 ± 2	17 ± 2	13 ± 2	<0.01*
Ejection Fraction (%)	73 ± 5	77 ± 9	76 ± 15	70 ± 13	67 ± 14	45 ± 16	<0.01*
Impaired Ejection Fraction (%)	0	0	0	15	14	100	<0.01*
Aortic valve area (cm^2^)	0.85 ± 0.30	0.90 ± 0.43	1.10 ± 0.32	0.98 ± 0.34	0.86 ± 0.25	0.80 ± 0.16	0.22
Peak Velocity (m/s)	3.6 ± 0.4	3.6 ± 0.8	3.42 ± 0.67	4.0 ± 0.97	3.80 ± 0.76	3.8 ± 0.8	0.17
Severe AS (%)	73	64	45	65	78	100	0.13
CLINICAL DATA							
Bicuspid valve (%)	55	45	27	41	29	40	0.76
Hypertension (%)	9	18	64	38	64	50	0.03*
Diabetes Mellitus (%)	18	0	18	15	7	30	0.45
ACEi/ARB (%)	20	10	55	39	36	22	0.18
Beta blocker (%)	20	10	37	18	18	20	0.56

Demographic, CMR and clinical data for patients with normal LV structure, concentric remodeling, asymmetric remodeling, concentric hypertrophy, asymmetric hypertrophy and LV decompensation.

### Determinants of left ventricular hypertrophy

**Figure 2:**
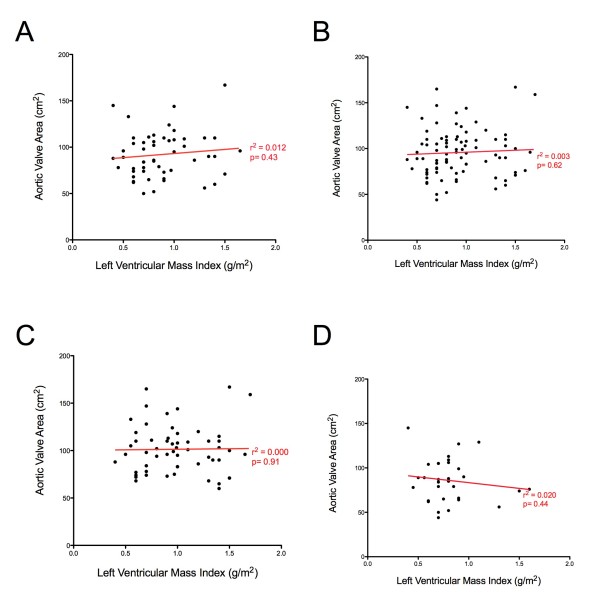
** Lack of correlation between aortic valve area and left ventricular mass index.A**. Total population. **B**. Population after excluding patients with hypertension. **C**. Males. **D**. Females.

The LV mass index was unrelated to aortic stenosis severity both in terms of the aortic valve area (r^2^ = 0.012 P = 0.43, Figure 2) and the aortic valve area indexed to body surface area (r^2^ = 0.002, P = 0.67). There was no difference in the degree of hypertrophy between patients with moderate and severe disease (mean difference in mass 3.9 g/m^2^, 95% CI -7.6 to 15.5 g/m^2^, P = 0.50). The lack of correlation between aortic stenosis severity (AVA) and the LV mass index persisted in sub-group analyses of sex (male: r^2^ = 0.000, P = 0.91; female: r^2^ = 0.020, P = 0.44) and after excluding those with hypertension (r^2^ = 0.003, P = 0.62; Figure 2).

After univariate analysis, male sex was the only variable associated with an increased LV mass index, being 13.8 g/m^2^ (95% CI, 2.8 to 24.7 g/m^2^, P = 0.02) higher in men (Table 2) with an apparent trend to an increased mass with co-existent hypertension (mean difference 9.9 g/m^2^; 95% CI, -1.1 to 20.9 g/m^2^, P = 0.08).

**Table 2:** Univariate analysis of the association between the indexed left ventricular mass and different independent variables

**Variable**	**Mean difference in Indexed Mass(g/m**^**2**^**)**	**Confidence Intervals**	**P value**
Age >66 years	7.5	-3.4–18.4	0.17
Male	13.8	2.8–24.7	0.02
Moderate Aortic Stenosis	3.9	-7.6–15.5	0.50
Bicuspid	-7.3	-18.4–3.9	0.20
Hypertension	9.9	-1.1–20.9	0.08
Diabetes mellitus	11.9	-3.6–27.4	0.13
ACE Inhibitor/ARB	11.2	-1.00–23.3	0.07
β-Blocker	3.2	-11.2–17.6	0.66

Male sex was the only variable associated with a significant increase in the LV mass index, being 13.8 g/m^2^ higher in males than females. There was no difference in LV mass between patients with moderate and severe aortic stenosis.

### Left ventricular characteristics of patients with aortic stenosis

Twelve per cent (n = 11) of subjects had normal left ventricular structure and 24% left ventricular remodeling (n = 22; 11 asymmetric and 11 concentric) (Table 1, Figure 1). Left ventricular hypertrophy was the most prevalent pattern and occurred in 53% (n = 48) of subjects (concentric 71%; asymmetric 29%). Absolute wall thickness was similar between patients with remodeling and hypertrophy (15 ± 3 *vs* 16 ± 2 mm; P = 0.16) but the indexed mass was significantly higher in those with hypertrophy (112 ± 22 vs 76 ± 9 g/m^2^; P < 0.001) and the LVEDV lower in those with remodeling (78 ± 20 v*s* 56 ± 10 ml/m^2^; P < 0.001). In the majority of patients with hypertrophy, left ventricular ejection fraction was preserved but it was impaired in 7 subjects (mean ejection fraction of 48%). No patient met the pre-defined criteria for eccentric hypertrophy. However, 10 patients (11%), all with severe AS, were observed to have a dilated left ventricle, increased indexed mass and normal M/V in the context of an impaired ejection fraction (45 ± 16%; p < 0.001 *vs.* normal group). This pattern was termed LV decompensation and replaced eccentric hypertrophy as the sixth pattern of LV adaption in our classification.

There was no relationship between aortic stenosis severity and the pattern of LV remodeling or hypertrophy (P = 0.22; Table 1). Compared to normotensive patients, those with co-existent hypertension were more likely to have an asymmetric pattern of wall thickening (43% vs. 17%, P = 0.01) but importantly there was no difference in the proportion of patients with LV remodeling (24% vs. 24%, P = 1.00), hypertrophy (59% vs. 48%, P = 0.39) or decompensation (14% vs. 9%, P = 0.73; Table 3).

**Table 3:** Comparison of the remodeling and hypertrophic response in aortic stenosis patients with and without concomitant hypertension

	**Hypertension**	**No Hypertension**	**P value**
Number	37	54	-
Male Sex %	23 (62%)	34 (62%)	1.00
Age (years)	70 ± 13	55 ± 21	<0.01*
Aortic Valve Area (cm^2^)	1.00 ± 0.33	0.88 ± 0.31	0.09
Indexed LVEDV (ml/m^2^)	76 ± 24	78 ± 29	0.74
LV mass index (g/m^2^)	102 ± 28	93 ± 25	0.08
M/V (g/ml)	1.41 ± 0.37	1.27 ± 0.38	0.10
Maximal wall thickness (mm)	16 ± 3	14 ± 3	<0.01*
Asymmetric Wall Thickening n (%)	16 (43%)	9 (17%)	0.01*
Pattern of Remodeling/Hypertrophy			
Normal n (%)	1 (3%)	10 (19%)	0.02*
Remodeling n (%)	9 (24%)	13 (24%)	1.00
Concentric n (%)	2 (5%)	9 (17%)	0.19
Asymmetric n (%)	7 (19%)	4 (7%)	0.11
Hypertrophy n (%)	22 (59%)	26 (48%)	0.39
Concentric Hypertrophy n (%)	13 (35%)	21 (39%)	0.83
Asymmetric Hypertrophy n (%)	9 (24%)	5 (9%)	0.07
LV Decompensation n (%)	5 (14)	5 (9%)	0.73

Categorical variables expressed as n (%) and compared using Fisher’s exact test. Continuous variables expressed as the mean ± SD and compared using the Student’s unpaired *t*-test. *P < 0.05.

### Asymmetric versus concentric patterns of wall thickness

**Figure 3:**
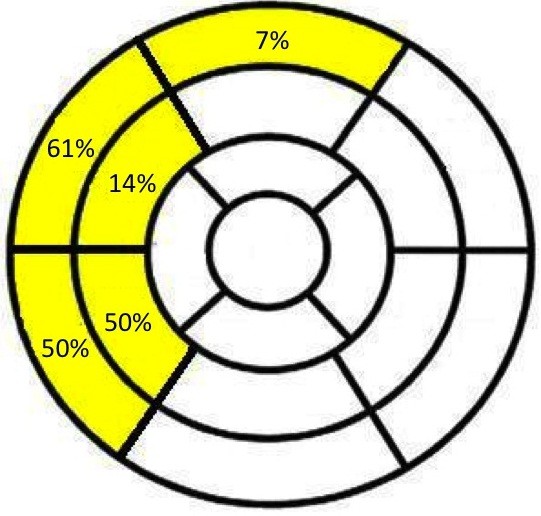
**Site of maximal wall thickening in asymmetric hypertrophy and remodeling based on the 17-segment model of the left ventricle.** Asymmetric wall thickening was observed in the basal anterior wall in 7%, otherwise it was confined to the septum at the basal and mid-cavity levels.

An asymmetric pattern of LV wall thickness was observed in 25 patients (27%) with remodeling or hypertrophy (Table 4). Compared to concentric patterns, patients were older (72 ± 11 *vs.* 56 ± 19 years, P < 0.001), more likely to have hypertension (64 *vs.* 33%, P = 0.01) and had a greater maximal wall thickness (17 ± 2 *vs.* 15 ± 3 mm, P < 0.001). The site of asymmetry was most often in the basal septum (Figure 3) and affected two segments of the 17-segment model in 72% of subjects (1 segment in 24%; 3 segments in 4%). In 7%, it affected the basal anterior wall but otherwise focal wall thickening was not observed outwith the septum.

**Table 4:** Comparison of patient characteristics between those with asymmetric and concentric forms of hypertrophy and remodeling in aortic stenosis

	**Concentric**	**Asymmetric**	**p-value**
Number	45	25	-
Age (years)	56 ± 19	72 ± 11	<0.01*
Male sex (%)	62	68	0.41
LV mass index (g/m^2^)	103 ± 25	96 ± 25	0.23
Max wall thickness (mm)	15 ± 3	17 ± 2	<0.01*
Hypertrophy (%)	76	56	0.09
Aortic valve area (cm^2^)	0.96 ± 0.36	0.96 ± 0.30	0.83
Ejection Fraction	72 ± 12	71 ± 15	0.79
Indexed LVEDV (mL/m^2^)	72 ± 20	69 ± 22	0.57
Hypertension (%)	33	64	0.01*
Diabetes Mellitus (%)	11	12	0.91
ACE inhibitor/ARB use (%)	33	45	0.45
Beta-blocker (%)	16	27	0.32

Asymmetric left ventricular wall thickening was defined as a regional wall thickening ≥13 mm that was >1.5-fold the thickness of the opposing myocardial segment. Criteria had to be fulfilled on two adjacent short-axis slices.

## Discussion

This is the first study to use CMR to investigate both the magnitude of the hypertrophic response and the different morphological patterns of remodelling and hypertrophy observed in patients with aortic stenosis. We have demonstrated that the degree and pattern of hypertrophy is independent of the severity of valve stenosis severity. We observed six distinct patterns of left ventricular adaption, which in line with previous echocardiographic definitions included normal left ventricular structure, concentric remodeling, and concentric hypertrophy. However in contrast to previous classifications, we also describe asymmetric remodeling and asymmetric hypertrophy as common structural variants.

### Magnitude of the hypertrophic response

The magnitude of left ventricular hypertrophy varied widely but was unrelated to the severity of aortic stenosis, such that patients with severe valve narrowing were found to have normal ventricular structure whilst patients with moderate disease often had extensive hypertrophy. This observation is consistent with the findings of several previous echocardiographic studies [18-20], but has not previously been reported using CMR.

The apparent disconnect between processes in the valve and myocardium might explain the marked heterogeneity between the severity of valve stenosis and symptom onset, and is important because an advanced hypertrophic response is associated with an adverse prognosis in a range of cardiac conditions including aortic stenosis [3-5,21]. Recent data has suggested that this might reflect its association with mid-wall fibrosis: an independent predictor of all-cause mortality in patients with moderate and severe disease [22]. We therefore believe that when considering overall aortic stenosis severity, attention should be paid not only to the degree of valve narrowing but also to the hypertrophic response accompanying it.

Consistent with previous studies, male gender was the only variable associated with an increased left ventricular mass [23,24] although an apparent trend was observed with concomitant hypertension. The latter was also seen in an analysis of the Simvastatin and Ezetimibe in Aortic Stenosis (SEAS) trial where hypertension predicted increased left ventricular mass in patients with aortic stenosis independent of other known confounders [25]. Strict blood pressure control may therefore provide an important clinical means of blunting the hypertrophic response.

Importantly the lack of correlation between valve stenosis and the hypertrophic response persisted in subgroup analyses of our male, female and normotensive patients indicating that gender and blood pressure cannot in themselves explain this observation. Genetic factors were not investigated in this study and are likely to play an important role. These are known to modulate the magnitude of the hypertrophic response to a number of physiological and pathological triggers [26-28]. These include aortic stenosis, in which polymorphisms of the angiotensin-converting enzyme I/D genotype have been associated with different degrees of wall thickening and hypertrophy [23], as well as the regression of these processes following valve replacement [29].

### Asymmetric remodeling and hypertrophy

An asymmetric pattern of wall thickening was observed in 27% of our cohort, being particularly prevalent amongst the elderly and in those with hypertension. This confirms the echocardiographic findings of Tuseth and colleagues who described asymmetric wall thickening in 22% of patients with aortic stenosis, and also observed an association with co-existent hypertension [9]. Importantly our study demonstrated asymmetric wall thickening in a sixth of normotensive patients with aortic stenosis, confirming that it is not only a response related to high blood pressure.

Asymmetric wall thickening was most frequently observed in the septum at the basal and mid-cavity levels with a mean of 17 mm and a maximum of 22 mm. Current guidelines recommend that a diagnosis of hypertrophic cardiomyopathy be considered if regional wall thickness exceeds 15 mm, with an intermediate area existing between 13 and 15 mm [30]. Our data therefore suggest considerable overlap in the appearances of asymmetric wall thickening in these two conditions and underlines the fact that the morphological diagnosis of hypertrophic cardiomyopathy may be challenging or impossible in the context of an increased afterload. Furthermore it is plausible that specific genotypes, related to those causing hypertrophic cardiomyopathy, may predispose to an asymmetric rather than concentric remodeling response. In line with this theory, patients with hypertension and asymmetric thickening have a higher familial incidence of hypertrophic cardiomyopathy and more myocardial disarray [31].

### Left ventricular decompensation

Eccentric hypertrophy has been included in previous definitions of remodeling and hypertrophy in aortic stenosis but this was not observed in our study [7]. The strict selection criteria employed in our population excluded patients with conditions such as aortic and mitral regurgitation and ischemic heart disease that in clinical practice will result in a composite form of left ventricular adaption and perhaps explain the eccentric phenotype observed in previous cohorts. Instead we observed a form of left ventricular decompensation characterized by impaired systolic function and left ventricular dilatation. This form of remodeling can be considered as the end-stage of the hypertrophic process in which the left ventricle has failed in the face of an increased afterload. Given that a reduction in ejection fraction is a powerful prognostic marker in aortic stenosis, this pattern of LV adaption is likely to be associated with an increased mortality [32]. Interestingly not all patients with a reduction in ejection fraction conformed to this phenotype: 41% had either concentric or asymmetric hypertrophy. It is likely that these patients are in the early stages of decompensation without having yet proceeded to left ventricular dilatation. They represent an important group to identify because prompt surgery might avoid a further deterioration in ejection fraction.

### Patterns of remodeling and aortic stenosis

It has been proposed that in response to an increased afterload patients progress from a normal ventricle to LV remodeling, and then hypertrophy before decompensating and developing heart failure. However in our study, there was no clear correlation between the severity of aortic valve narrowing and the different patterns of LV adaption, except that all patients with LV dilatation had severe disease. This may simply reflect the relatively small sample size, however there are two alternative explanations. Firstly patients transition through the various stages of the remodeling process at different rates, much in the same way that they display variation in the magnitude of the hypertrophic response. Secondly patients do not in fact progress with time from LV remodeling to hypertrophy but instead these represent two distinct and independent pathways of adaption. Prospective longitudinal studies are required to address this issue.

### Study limitations

Thirty-seven patients from our cohort had co-existent mild to moderate hypertension. Given that hypertension and aortic stenosis commonly co-exist, it was not desirable to exclude all patients with hypertension from the study, as this would have affected the generalizability of our findings. However, sub-group analysis allowed us to examine how co-existent hypertension might modulate the effect of valve narrowing. This demonstrated that hypertension was associated with an apparent increase in the LV mass index and an increased proportion of patients with asymmetric wall thickening. Importantly however the lack of correlation between the LV mass index and aortic stenosis severity remained and was independent of the effects of blood pressure.

In our institution local guidelines recommend CMR for all patients with severe aortic stenosis. However patients with moderate disease were referred at the discretion of their clinician and therefore there may have been some referral bias in this group. In addition, we have not examined patients with mild disease nor the influence of the duration of aortic stenosis. The latter is almost impossible to adjudicate because the majority of patients will have subclinical disease for many years before a murmur is detected and the diagnosis established.

Multi-centre longitudinal trials are required in an unbiased population to confirm our findings and to provide prognostic information. In the era of transcatheter aortic valve implantation (TAVI), where earlier treatment may have benefit, it will also be important to assess if the different patterns of remodeling and hypertrophy show variable potential for reverse remodeling following intervention.

## Conclusions

We have demonstrated marked variation in the hypertrophic response of the left ventricle in a selected cohort of patients with aortic stenosis in the absence of coronary artery disease, cardiomyopathy and other forms of valve disease. The degree of the hypertrophic response was independent of the severity of valve narrowing and six distinct patterns of LV adaption were observed. These included asymmetric remodeling and hypertrophy, which displayed considerable overlap in appearances with hypertrophic cardiomyopathy. This variation highlights the importance of detailing the left ventricular remodeling response in all patients with aortic stenosis, particularly given the adverse prognosis associated with increased levels of hypertrophy.

## Abbreviations

AS, Aortic Stenosis; AVA, Aortic Valve Area; CMR, Cardiovascular Magnetic Resonance; LV, Left Ventricular; LVEDV, Left Ventricular End Diastolic Volume; TAVI, Transcatheter Aortic Valve Implantation.

## Competing interests

The authors declare that they have no competing interests.

## Authors’ contributions

MRD devised the idea for the project and led the data analysis and the drafting and redrafting of the manuscript. SJ, TM AG, FA, AJ, AM IR were involved in the image and data analysis. DBN, SAC, NAB, JRP, RHM and DEN helped develop the idea for the project and provided considerable editorial assistance in drafting the manuscript. SKP and DJP were responsible for creating the initial registry, and provided major assistance with the development of the project and editing of the manuscript. All authors read and approved the final manuscript.
